# NGmerge: merging paired-end reads via novel empirically-derived models of sequencing errors

**DOI:** 10.1186/s12859-018-2579-2

**Published:** 2018-12-20

**Authors:** John M. Gaspar

**Affiliations:** Informatics Group, Division of Science, Harvard University Faculty of Arts and Sciences, Cambridge, MA 02138 USA

**Keywords:** High-throughput sequencing, Illumina paired-end sequencing, Read merging, sequencing errors; quality scores; PhiX

## Abstract

**Background:**

Advances in Illumina DNA sequencing technology have produced longer paired-end reads that increasingly have sequence overlaps. These reads can be merged into a single read that spans the full length of the original DNA fragment, allowing for error correction and accurate determination of read coverage. Extant merging programs utilize simplistic or unverified models for the selection of bases and quality scores for the overlapping region of merged reads.

**Results:**

We first examined the baseline quality score - error rate relationship using sequence reads derived from PhiX. In contrast to numerous published reports, we found that the quality scores produced by Illumina were not substantially inflated above the theoretical values, once the reference genome was corrected for unreported sequence variants. The PhiX reads were then used to create empirical models of sequencing errors in overlapping regions of paired-end reads, and these models were incorporated into a novel merging program, NGmerge. We demonstrate that NGmerge corrects errors and ambiguous bases better than other merging programs, and that it assigns quality scores for merged bases that accurately reflect the error rates. Our results also show that, contrary to published analyses, the sequencing errors of paired-end reads are not independent.

**Conclusions:**

We provide a free and open-source program, NGmerge, that performs better than existing read merging programs. NGmerge is available on GitHub (https://github.com/harvardinformatics/NGmerge) under the MIT License; it is written in C and supported on Linux.

**Electronic supplementary material:**

The online version of this article (10.1186/s12859-018-2579-2) contains supplementary material, which is available to authorized users.

## Background

Among the high-throughput DNA sequencing technologies, the Solexa/Illumina platform [1] produces the greatest quantity of sequence data in a single run [2]. One unique attribute of this technology is its ability to generate sequence reads from both ends of a given DNA molecule. This provides many opportunities for biological interpretation; for example, one can infer the full extent of a DNA molecule without sequencing its entirety, by aligning the paired-end reads to a reference sequence.

The output from an Illumina sequencing run is a set of FASTQ files, which contain read sequences and corresponding quality scores [3]. As first developed for Sanger sequencing, a quality score is determined from the probability that a given sequenced base is wrong, via the following equation [4]:

1 $$ {\mathrm{Q}}_{\mathrm{base}}=-10\times {\log}_{10}\left(\mathrm{P}\left(\mathrm{base}\kern0.5em \mathrm{wrong}\right)\right) $$

Thus, a base with a quality score of 40 should have a 1 in 10,000 chance of being wrong. In sequence variant detection, many programs will consider only bases that achieve a minimum quality score, in order to reduce false positives [5], and in clinical variant detection, such as cancer diagnostics, published guidelines frequently incorporate such standards [6, 7]. However, numerous studies have shown that the raw quality scores produced by Illumina machines are inflated; that is, sequenced bases with a given quality score have higher error rates than expected from Eq. (1), especially at the high end of the scale [8–11].

Advances in Illumina sequencing technology have given rise to reads of increasing length, such that the paired-end reads for a particular library may have substantial sequence overlaps. Since these overlapping regions do not represent independent sequence data, it is possible to merge the reads into a single read spanning the full length of the original DNA fragment. This merging process allows for error correction and accurate determination of read coverage, and it has become increasingly appreciated in applications ranging from targeted variant resequencing [12] to metabarcoding (e.g., 16S/18S rRNA studies) [13].

**Fig. 1:**
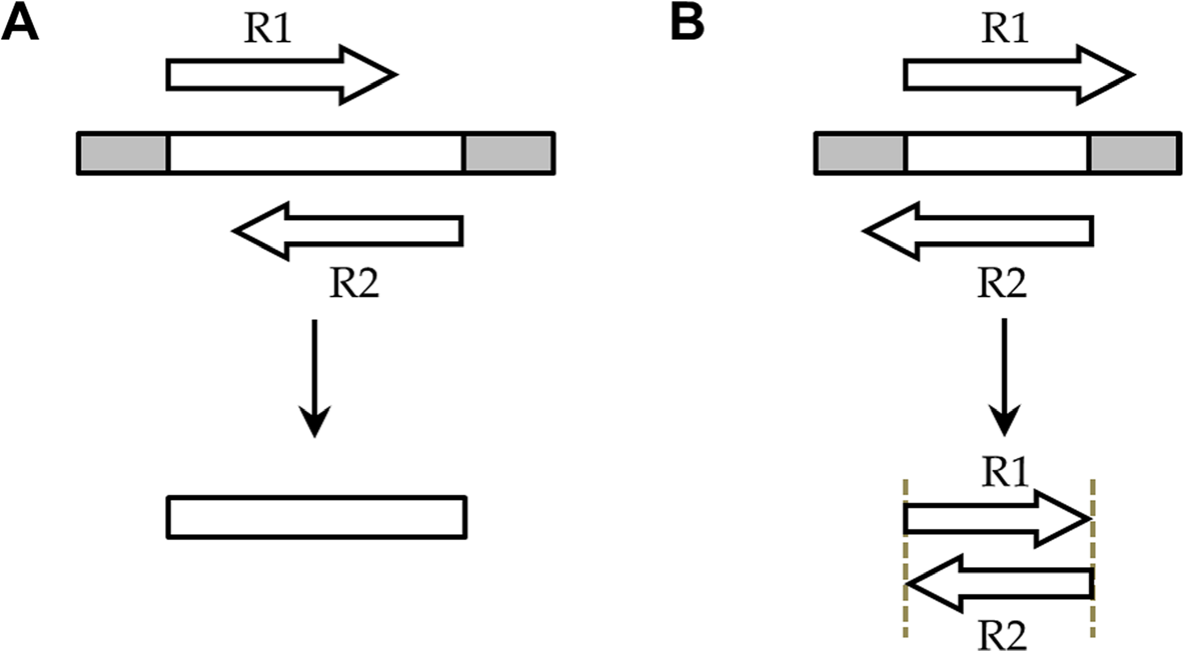
Analysis modes of NGmerge. The diagrams show the paired-end reads (R1, R2) derived from sequencing DNA fragments (white boxes) with sequencing adapters (gray boxes) on either end. **a** In the default mode (“stitch”), NGmerge combines paired-end reads that overlap into a single read that spans the full length of the original DNA fragment. **b** The alternative “adapter-removal” mode returns the original reads as pairs, removing the 3′ overhangs of those reads whose optimal alignment has this characteristic

An early merging program was fastq-join [14], which was the default option in the microbial ecology analysis package QIIME [15]. The latest version of that package, QIIME 2, uses the open-source VSEARCH [16]. A third merging program is PEAR [17], which has the significant advantage over the other two programs (and other programs such as FLASH [18] and CASPER [19]) of considering “dovetailed” alignments, in which one read’s 3′ end extends past its pair’s 5′ end (see Fig. 1b). The sequencing of DNA fragments that are shorter than the read lengths will result in reads that contain portions of sequencing adapters on their 3′ ends; such read pairs will not be merged by programs that fail to consider dovetailed alignments [17].

Another important difference among read merging programs is the method for assigning quality scores to the merged bases. Fastq-join and FLASH use a simplistic scheme in which, if the bases of the two reads match, the higher quality score is used for the merged base. Where the bases disagree, the base with the higher quality score is selected, and the difference in quality scores becomes the merged base’s score. PEAR was designed with the reasoning that, if the bases of the original R1 and R2 reads agree, then the quality score of the merged base should reflect the probability that both original bases were wrong:

2 $$ {\mathrm{Q}}_{\mathrm{merged}\ \mathrm{base}}=\hbox{-} 10\times {\log}_{10}\left(\mathrm{P}\left(\mathrm{R}1\kern0.5em \mathrm{base}\kern0.5em \mathrm{wrong}\kern0.5em \mathrm{AND}\kern0.5em \mathrm{R}2\kern0.5em \mathrm{base}\kern0.5em \mathrm{wrong}\right)\right) $$

3 $$ {\mathrm{Q}}_{\mathrm{mergedbase}}=-10\times {\log}_{10}\left(\mathrm{P}\left(\mathrm{R}1\kern0.5em \mathrm{base}\kern0.5em \mathrm{wrong}\right)\times \mathrm{P}\left(\mathrm{R}2\kern0.5em \mathrm{base}\kern0.5em \mathrm{wrong}\right)\right) $$

4 $$ {\mathrm{Q}}_{\mathrm{merged}\ \mathrm{base}}=\hbox{-} 10\times {\log}_{10}\left(\mathrm{P}\left(\mathrm{R}1\ \mathrm{base}\ \mathrm{wrong}\right)\right)+\hbox{-} 10\times {\log}_{10}\left(\mathrm{P}\left(\mathrm{R}2\ \mathrm{base}\ \mathrm{wrong}\right)\right) $$

5 $$ {\mathrm{Q}}_{\mathrm{merged}\ \mathrm{base}}={\mathrm{Q}}_{\mathrm{R}1}+{\mathrm{Q}}_{\mathrm{R}2}\kern1em $$

Thus, PEAR sums quality scores for matching bases. VSEARCH utilizes a more sophisticated model developed by Edgar and Flyvbjerg [20], but the resulting scheme for matching bases is nearly identical to that of PEAR (see Additional file 1: Figure S1). For example, a merged base created from matching bases with quality scores of 40 would be assigned a quality score of 40 by fastq-join, 80 by PEAR, and 85 by VSEARCH (ignoring the artificial caps on quality scores placed by the programs).

None of these quality score profiles has been tested empirically, despite possible shortcomings. For example, the profiles of PEAR and VSEARCH are based on Eq. (1), which, as noted above, has been demonstrated to be inaccurate with Illumina sequencing. Furthermore, both PEAR and VSEARCH were designed under the assumption that sequencing errors in the two reads are independent; that is, in the analysis above, Eq. (3) follows from Eq. (2) only if the two events (“R1 base wrong” and “R2 base wrong”) are independent. This assumption has, to our knowledge, never been verified.

In this manuscript, we first evaluate the baseline quality score - error rate relationship produced by Illumina machines using reads derived from the enterobacteria phage ΦX174 (“PhiX”). This virus’ genome was the first DNA genome to be sequenced [21], and a library composed of fragments of PhiX DNA is routinely added to Illumina sequencing runs as a control. In addition to the wide accessibility of PhiX reads, the sizes of the fragments in this library are such that most PhiX-derived read pairs produced by longer Illumina runs will have sufficient overlaps that can be used to create quality score profiles for merged bases (Additional file 1: Figure S2). We have done this and incorporated these profiles into a novel merging program, NGmerge (Fig. 1). We demonstrate that NGmerge corrects errors and ambiguous bases (Ns) better than other merging programs, and produces merged reads whose quality scores accurately reflect the bases’ error rates.

## Results

### Baseline error rates

We began with nine Illumina sequencing runs that yielded 2 × 250 bp paired-end reads, produced at Harvard University. After identifying reads that originated from PhiX, we calculated error rates for each quality score. Consistent with previous studies [8–11], the error rates were higher than expected based on Eq. (1) above; bases with quality score 40 had error rates an order of magnitude above the predicted 1 × 10^− 4^.

However, a closer look at the alignments revealed variants from the canonical PhiX reference genome. In all of the sequencing runs, the same five sequence variants were identified at a minimum 95% allele frequency, with most at greater than 99% (Table 1). No other variants were identified.

**Table 1:** Variants in the PhiX genome

Position (1-based)	NCBI base	iGenomes base	Observed base
587	G	A	A
833	G	A	A
2731	A	G	G
2793	C	T	C
2811	C	T	T
3133	C	C	T

Four of the five observed variants are in the version of the PhiX genome provided by Illumina on its iGenomes website (https://support.illumina.com/sequencing/sequencing_software/igenome.html; retrieved Nov. 2017), and it lists an additional variant at position 2793 that was not observed in any sequencing run. Nevertheless, the iGenomes version is considered current by Illumina (personal communication, Nov. 2017)

**Fig. 2:**
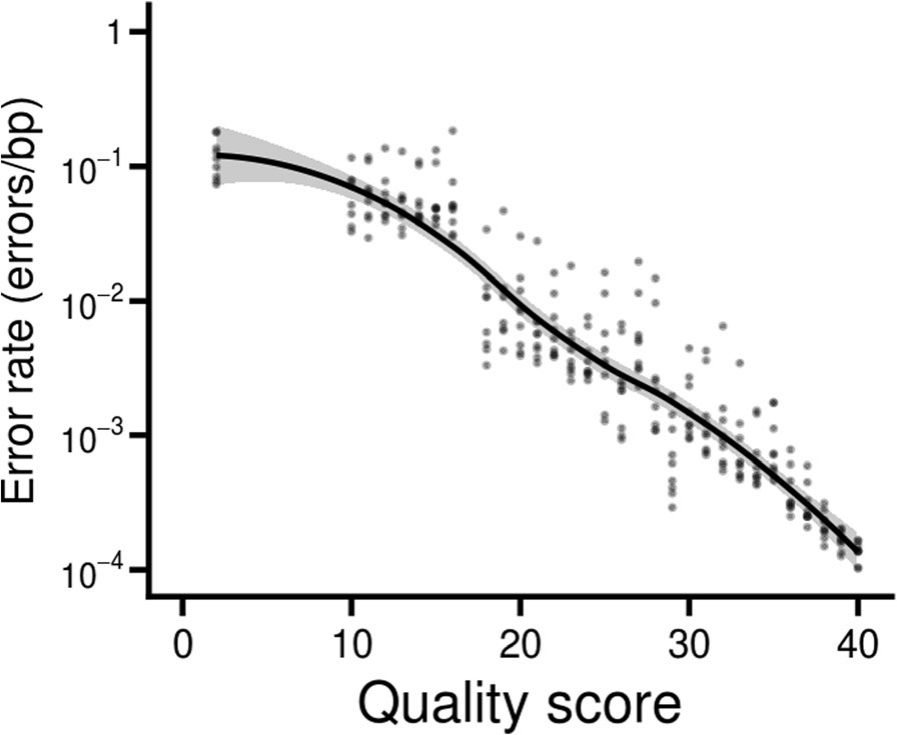
Error rate - quality score relationship. The quality scores of the original paired-end reads in the Harvard datasets followed a nearly linear relationship with the log of the error rates, consistent with expectations

We modified the reference genome to incorporate the five observed variants. Once this was done, the error spectrum more closely matched the expected relationship (Fig. 2). For example, bases with a quality score of 40 had error rates whose average corresponded to a true value of 38.6. The major deviation was at the low end of the scale, where bases with quality scores of two had considerably lower error rates than expected (Fig. 2).

### Creating quality score profiles

**Fig. 3:**
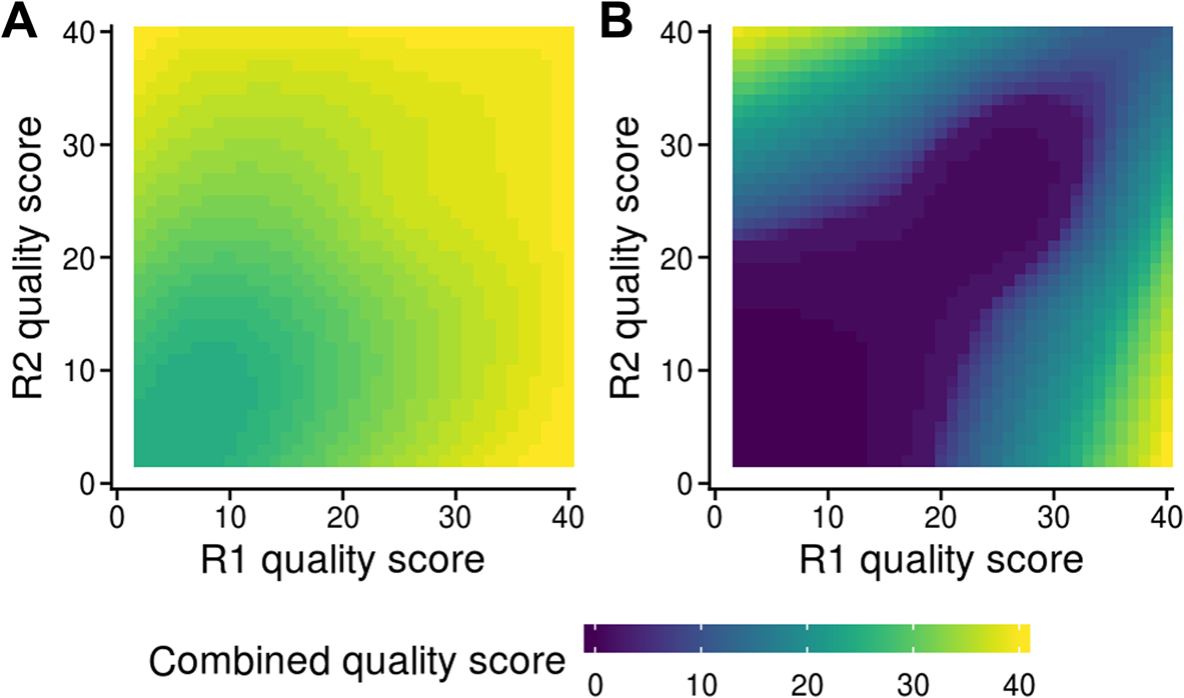
Quality score profiles of NGmerge. **a** A plot of the quality scores corresponding to the error rates calculated for each combination of the two reads’ quality scores, for cases where the bases matched. **b** Same as A, but for cases where the bases of the paired reads did not match

Again using the Harvard datasets, we computed error rates in regions where the paired reads overlapped each other, for each possible combination of the two reads’ quality scores. We then converted these rates back into quality scores, using the baseline error rate already calculated (Fig. 2). In cases where the bases of the two reads agreed (Fig. 3a), which amounted to 96.7% of all overlapping bases, no combinations yielded scores below 25, even where both reads had low-quality bases. However, two high-quality matching bases did not produce substantially increased quality scores, with no combined scores rising above 40. This contrasts with the scoring schemes of VSEARCH and PEAR, which assign scores of up to 85 and 80, respectively (Additional file 1: Figure S1).

On the other hand, where the two reads’ bases disagreed (Fig. 3b), the combined quality scores were lowest when the two original quality scores were similar to each other, and rose above 30 only when the two quality scores were at opposite ends of the scale. This is similar to the schemes of fastq-join and VSEARCH, whereas PEAR does not reduce quality scores for mismatches (Additional file 1: Figure S1).

These two quality score models were incorporated into our merging program, NGmerge, to be utilized in the creation of merged reads.

### Comparing merging programs

To compare the performance of NGmerge against other merging programs, we opted not to use the same Harvard datasets, since they had trained NGmerge’s models. Instead, we queried the Sequence Read Archive (SRA) for datasets that had sufficient read lengths and PhiX content to be usable for calculating error rates. We found 33 such datasets, containing a total of 1386 sequencing runs (see Methods and Additional file 2: Table S1 for details).

First, we determined that each of the SRA datasets had the same five PhiX genomic sequence variants previously identified (Table 1). In addition, the baseline error rates of the datasets’ reads followed a similar trend to those of the training datasets. We then processed the datasets through each of the merging programs (since neither fastq-join nor VSEARCH consider dovetailed alignments, the datasets were preprocessed by NGmerge in adapter-removal mode (Fig. 1b) prior to analysis with those programs).

**Fig. 4:**
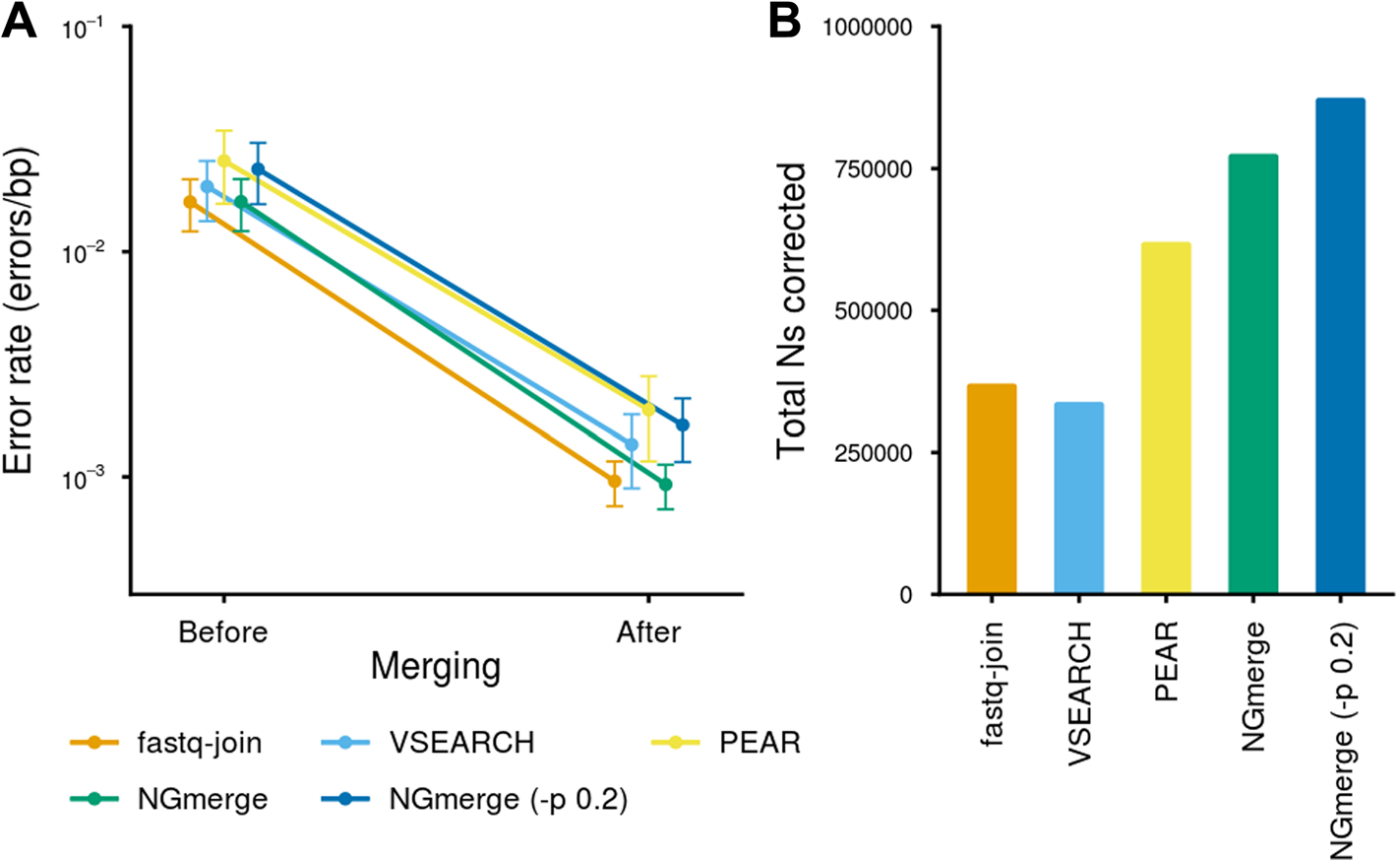
Errors and Ns corrected by the merging programs. **a** Error rates in the paired reads’ overlap regions, before and after the application of the merging programs. Note that the “Before” error rates vary because different merging programs analyze slightly different sets of reads (see Additional file 2: Table S1). **b** Total number of Ns corrected by each of the merging programs

All of the programs reduced the total error rates in the overlapping regions of the reads of the SRA datasets (Fig. 4a, Additional file 1: Table S2), with NGmerge producing the lowest rate, slightly better than fastq-join. The error rate after PEAR was more than twice those of the others, due to PEAR’s more aggressive merging algorithm causing a higher starting value. Increasing the fraction mismatch parameter of NGmerge (-p 0.2) led to merging results similar to those of PEAR, though with a lower final error rate (Fig. 4a, Additional file 1: Table S2). However, we found that the program CASPER produced an even lower error rate than NGmerge on a subset of the datasets, due to CASPER’s reliance on k-mer based contexts to resolve mismatches.

In addition to errors, sequence reads sometimes contain ambiguous bases (Ns), which can also complicate downstream analyses. Though ambiguous bases comprised just ~ 0.03% of overlapping bases in the SRA datasets, NGmerge’s unique approach (counting them as neither matches nor mismatches during alignment) led to the correction of the most, twice the counts of fastq-join and VSEARCH, and 25% more than PEAR (Fig. 4b).

**Fig. 5:**
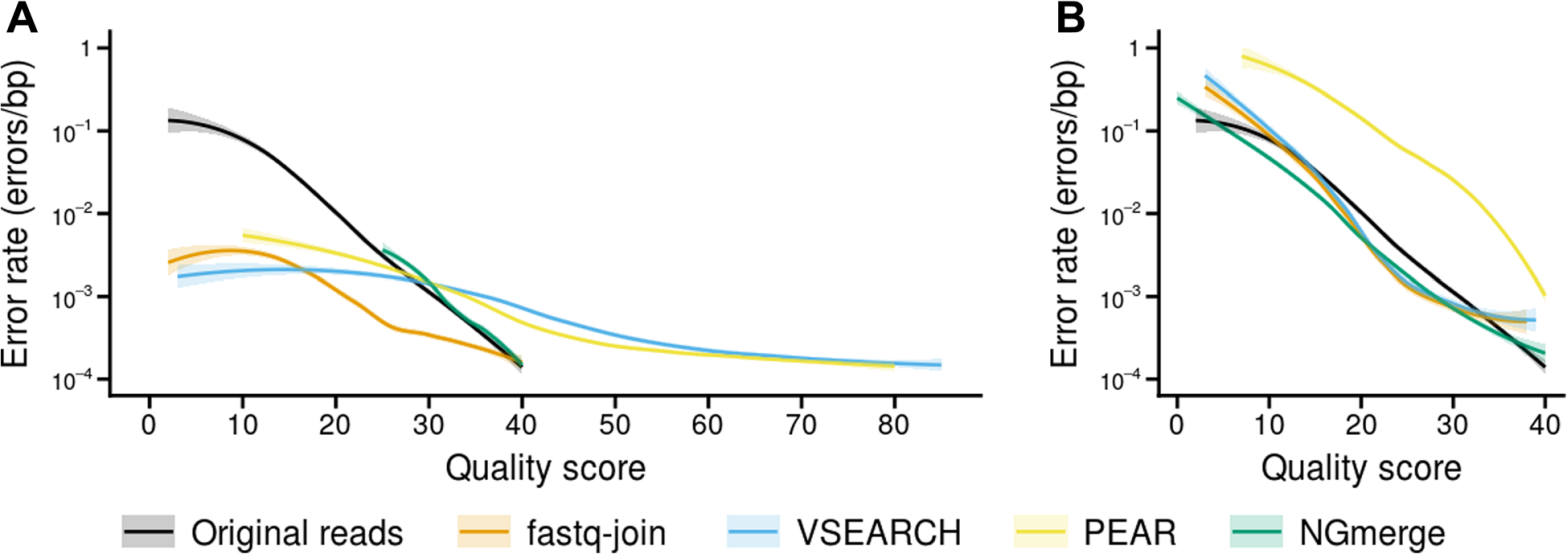
Error rate - quality score profiles produced by the merging programs. **a** Comparison of the profiles when the overlapping bases of the reads matched. The black line represents the baseline error rate - quality score profile of the original reads. **b** Comparison of the profiles when the overlapping bases did not match

We further examined the quality scores produced by each merging program. In cases where the overlapping reads’ bases matched (Fig. 5a), the error profile produced by NGmerge closely tracked that of the original reads. With the other three mergers, bases assigned quality scores below 28 had lower error rates than expected. However, both VSEARCH and PEAR greatly overstated the quality scores at higher values. For example, at a quality score of 80, VSEARCH and PEAR produced bases whose actual error rates were 1.8 × 10^− 4^ and 1.3 × 10^− 4^, respectively, more than four orders of magnitude above the theoretical value of 1 × 10^− 8^.

Where the original reads’ bases did not match (Fig. 5b), NGmerge slightly underestimated the quality scores throughout most of the score range. Fastq-join and VSEARCH followed similar paths, going above the baseline profile only at the ends of the quality score range. The merged bases produced by PEAR had far higher error rates than expected throughout the quality score range.

## Discussion

Of the merging programs analyzed, NGmerge has the best performance. It considers dovetailed alignments and thus does not require a separate adapter-removal step prior to merging reads; this more than compensates for its slightly worse run-time compared to VSEARCH (Additional file 1: Note S1). Furthermore, NGmerge produces lower error rates and corrects more Ns than the other programs. We note that NGmerge’s method for resolving mismatched bases may be improved by implementing a context-based scheme like that of CASPER. However, such an approach may have difficulty distinguishing sequencing errors from true biological variants in real samples; this is an area for further research.

In addition, NGmerge creates merged reads whose quality scores accurately reflect the bases’ error rates, unlike the other merging programs. It is noteworthy that the quality scores that deviate the most from the expected error rates are produced by VSEARCH and PEAR in merging matching bases (Fig. 5a). As explained above, these programs’ quality score calculations are based on the assumption of the independence of sequencing errors in paired reads [17, 20]. Our results demonstrate that this assumption is false. Therefore, the models produced by Edgar and Flyvbjerg [20] are invalid.

**Fig. 6:**
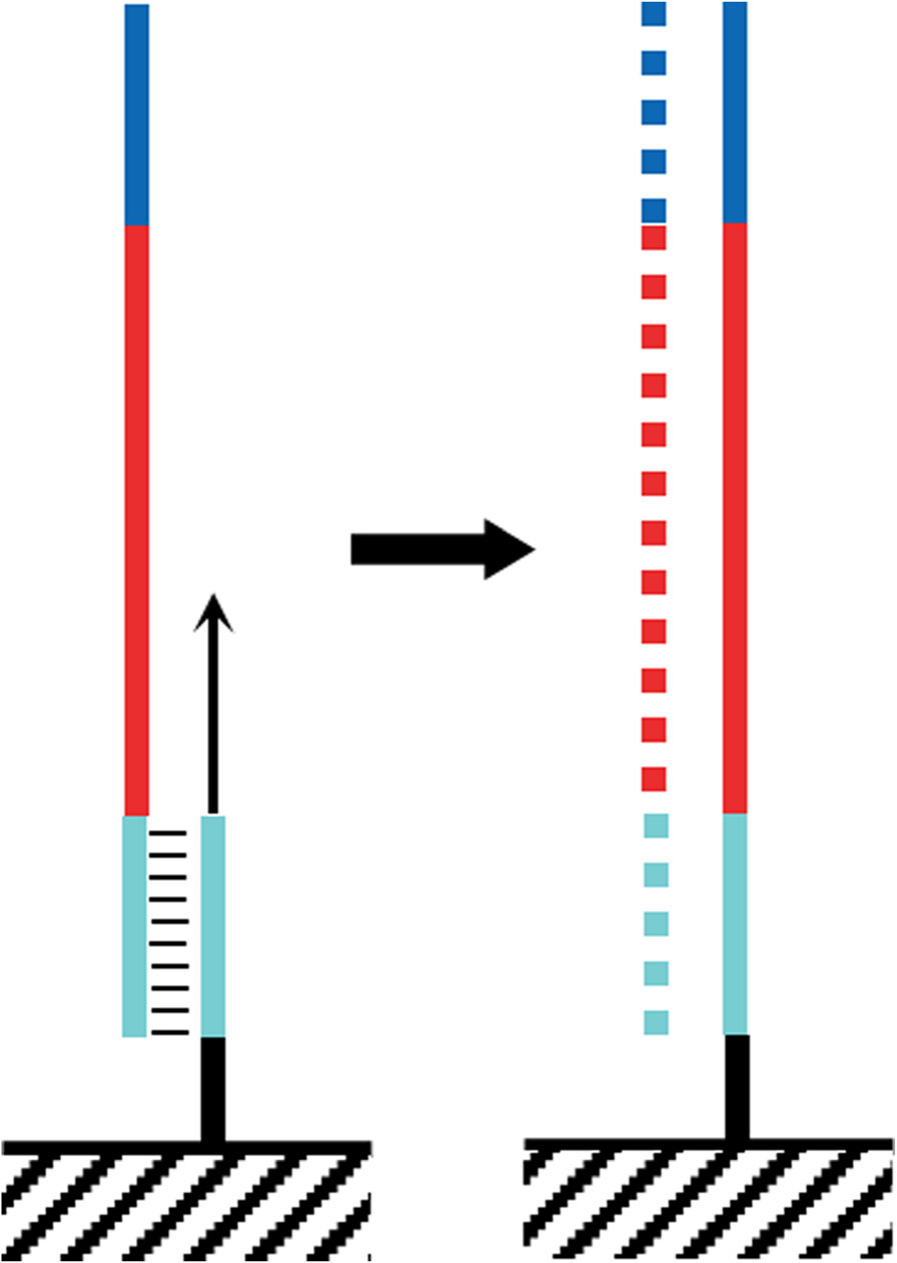
First-strand synthesis on the flow cell. A single-stranded DNA fragment to be sequenced anneals to an oligonucleotide that is covalently attached to the flow cell surface. The primer is extended to copy the DNA fragment, which is then removed by denaturation [1]

One reason for the lack of independence of errors in paired-end sequencing stems from the beginning of a sequencing run, during first-strand synthesis (Fig. 6). Since the original DNA fragment is denatured after it is copied, any errors made during this step will be propagated throughout the cluster that is formed during bridge amplification. Thus, both paired reads will contain the errors, but the erroneous bases will not have reduced quality scores.

Because of our reliance on reads derived from PhiX, NGmerge’s quality score profiles were tested only on datasets generated by MiSeq and HiSeq instruments. Thus, they may not work as well with other Illumina platforms, such as the NextSeq. NGmerge provides the option to forgo its default quality score profiles and instead to utilize calculations similar to those of fastq-join (and FLASH), which, though simplistic, are conservative over most of the score ranges. A third option is for the user to supply custom matrices of quality score profiles to NGmerge.

Although a number of studies have concluded that Illumina’s quality scores are substantially inflated, our results contradict this notion. Inaccuracies in reference sequences are a persistent problem that adversely affect error rate calculations [22], and in fact that proved to be the case here. Once the PhiX reference genome was corrected to account for the five sequence variants, the calculated error rates closely followed the expected relationship shown in Eq. (1). It is important to note that, in general, errors occurring during the library preparation process (e.g. PCR amplification) can be misconstrued as sequencing errors, leading to specious conclusions [23]. This is another reason why unamplified PhiX remains an enduring control in Illumina sequencing applications.

## Conclusions

We have examined errors produced by Illumina sequencing technology via reads derived from PhiX. We have found that variants from the canonical PhiX reference genome account for most of the discrepancy between the actual and theoretical relationships between quality scores and error rates. Furthermore, in the course of developing empirical models for error rates of paired-end sequence reads, we have demonstrated the fallacy of the assumption that has been repeatedly made, both implicitly and explicitly, that errors in such reads are independent.

Finally, we have described a free and open-source program, NGmerge, that merges paired-end sequence reads, thus correcting errors and ambiguous bases, and assigning quality scores that are consistent with the measured error rates. The program can also be run in an alternative mode simply to remove contaminating sequencing adapters. Complete descriptions of the usage and options of NGmerge are found on the homepage of the software (https://github.com/harvardinformatics/NGmerge) and in the accompanying UserGuide. The program is written in C and is parallelized with OpenMP 4.0.

## Methods

### NGmerge design

NGmerge operates on paired-end reads in two distinct modes, “stitch” and “adapter-removal” (Fig. 1). In either mode, NGmerge tests all possible gapless alignments of a pair of reads in attempting to find an optimal alignment. By default, NGmerge requires that a valid alignment have a minimum overlap of 20 bp and a maximum of 10% mismatches in the overlap region (-m 20 -p 0.1). If multiple valid alignments are found, the one with the lowest fraction mismatch is selected as the optimum. In all of these calculations, ambiguous bases (Ns) are considered neither matches nor mismatches. When the ‘-d’ option is set, or in adapter-removal mode, NGmerge will also attempt to align the reads in a dovetailed configuration (such as that shown in Fig. 1b), with 3′ overhangs corresponding to contaminating sequencing adapters that will be removed.

In stitch mode, NGmerge forms a single merged read that spans between the 5′ ends of the two original reads. The bases and quality scores of any non-overlapping regions are copied into the new read. For the overlapping region, if the bases of the R1 and R2 reads match, that base is used for the merged read, with the quality score determined from the “match” matrix (see “Harvard datasets” below). Where the bases disagree, the base with the higher quality score is selected, and the “mismatch” matrix yields the merged quality score.

### Merging programs

NGmerge (v0.2) was run with default alignment parameters, requiring a minimum overlap of 20 bp and a maximum of 10% mismatches. With the SRA datasets, it was also run allowing 20% mismatches (-p 0.2). The ‘-d’ option was set to allow for dovetailed alignments and the automatic removal of sequencing adapters.

Fastq-join (v1.01.759) [14] was run with alignment parameters analogous to those of the defaults of NGmerge (-m 20 -p 10). Because fastq-join does not allow for dovetailed alignments, adapters were removed from the reads with NGmerge prior to analysis with fastq-join.

VSEARCH (v2.6.2) [16], like fastq-join, does not consider dovetailed alignments, so it was also given reads from which adapters were removed with NGmerge. The minimum overlap length was increased to 20 bp (--fastq_minovlen 20). The maximum number of mismatches was greatly increased (--fastq_maxdiffs 30); even so, VSEARCH still analyzed the fewest reads (Additional file 2: Table S1). The cap on output quality scores was increased from the default value of 41 (--fastq_qmaxout 85).

PEAR (v0.9.10) [17] was run with a 20 bp minimum overlap (-v 20) and a maximum *p*-value threshold of 0.0001 (there is no fraction mismatch parameter). The cap on output quality scores was increased from the default value of 40 (-c 80).

With NGmerge, the arguments ‘-j <file> -b’ were specified so that the program would produce a file listing overlap mismatches and Ns, for later error counting. With the other merging programs, a custom Python script (findDiffs.py) reconstructed the alignments and determined the overlap mismatches.

Further details of these programs’ approaches toward read merging, along with an illustrative example, are provided in Additional file 1: Note S2.

### Calculation of error rates

The 5386-bp genome of the enterobacteria phage ΦX174, *sensu lato*, was retrieved from NCBI (accession NC_001422.1). The reads of each of the datasets were aligned to this genome using Bowtie2 [24], as described below. Pileup files were created from the alignment files using SAMtools (v1.5) mpileup (-B -Q 0 -d 1e9) [25], and variants were called with VarScan (v2.4.1) pileup2snp (--min-var-freq 0.15) [5].

The downloaded PhiX genome was modified to incorporate the five variants observed in the datasets (Table 1). Furthermore, because the PhiX genome is circular, a fragment corresponding to the first 1 kb (including the variants at positions 587 and 833) was appended to the end of the genome. This produced a final reference genome of 6386 bp that was used in all further analyses.

Reads were aligned to the modified PhiX reference genome using Bowtie2 (v2.3.2). The parameters of the program were modified to increase the strictness of accepted alignments, specifically by increasing the minimum score threshold (--score-min L,0,-0.2) and increasing gap penalties (--rdg 5,15 --rfg 5,15). The size of allowed fragments was increased to 1 kb (-X 1000), and the effort parameters were adjusted (--very-sensitive).

For the analyses of unmerged paired-end reads, contaminating sequencing adapters were removed by NGmerge (‘-a’ mode) prior to alignment. Only properly-paired alignments (‘samtools view -f 0x2’) were used to calculate error rates.

Error rates were calculated by quality score for the alignments in the SAM alignment files by a custom Python script (countErrors.py). When analyzing SAM files of merged reads, the script was provided the original read length(s) and a list of merging mismatches and Ns, in order to further categorize the errors based on the nucleotides in the R1 and R2 original reads, into matches, mismatches, and Ns (Additional file 1: Figure S3). The list of mismatches and Ns was produced by NGmerge or findDiffs.py, as described above.

In order to create the error profiles of NGmerge, we used the script countErrors3D.py, which tallied errors based on both original reads’ quality scores.

The three custom Python scripts are freely available on GitHub (https://github.com/jsh58/NGmerge/tree/master/scripts/).

### Harvard datasets

Nine sequencing datasets produced by the Bauer Core Facility of Harvard University, Faculty of Arts and Sciences Division of Science, between January 2016 and May 2017 were analyzed. Each sequencing run was produced on the Illumina HiSeq 2500 platform, yielding 2 × 250 bp paired-end reads. The reads placed into the “undetermined” bins were examined, a total of 553.0 million read pairs.

The paired-end reads were aligned to the modified PhiX genome after adapter-trimming with NGmerge, as described above. A LOESS regression function relating the quality scores to the logarithm (base 10) of the error rates was calculated in R (v3.4.1). This formed the baseline error profile for subsequent analyses.

To create the quality score profiles of NGmerge, the same reads were processed with NGmerge in stitch mode, allowing dovetailed alignments (-d). The merged reads were aligned to PhiX, and error rates were calculated for each combination of the quality scores of the R1 and R2 reads with countErrors3D.py. The “match” table, consisting of error rates for the locations where the bases of the two reads agreed, was edited to exclude values derived from fewer than 1000 counts, and values of zero were given a pseudo-error count of 0.5 (yielding a rate of 0.5/base count). These same edits were made to the “mismatch” table (error rates where the bases of the two reads disagreed), except that the minimum count threshold was lowered to 100 because of the reduced number of counts. Then, for each table, a two-dimensional LOESS regression function (relating both quality scores to the log (base 10) of the error rates) and predicted error rates were calculated in R. These error rates were then transformed back into quality scores using the baseline error profile calculated for the original paired-end reads. The resulting “match” and “mismatch” matrices were incorporated into NGmerge as the default quality score profiles.

### SRA datasets

The Sequence Read Archive (SRA) of NCBI (https://www.ncbi.nlm.nih.gov/sra) was queried for datasets containing paired-end reads that were minimum 2 × 250 bp in length. The sequencing runs of over 160 SRA studies were examined, though some were eliminated immediately for various reasons (mislabeled as paired-end; actual read lengths shorter than stated; reads already trimmed). The remaining datasets’ reads were adapter-trimmed with NGmerge and aligned to the PhiX genome. Those with at least 10,000 read pairs aligning to PhiX in a properly-paired configuration were further analyzed. The details of these 33 datasets, which contained 1386 sequencing runs and a total of 2.25 billion read pairs, are provided in Additional file 2: Table S1.

The reads of the 33 SRA datasets were analyzed in a similar fashion to the Harvard datasets. The baseline error rates were calculated from the original reads, and error rates were also determined after processing the reads with each of the merging programs. For each set of error rates, LOESS regression functions were computed, relating the quality scores to the log (base 10) of the error rates.

## Additional files

Additional file 1: **Figure S1.** Quality score profiles of the merging programs. **Figure S2.** PhiX library fragment lengths and paired-end read overlaps. **Figure S3.** Error rate calculation. **Table S2.** Error rates before and after merging. **Note S1.** Benchmarking of merging programs. **Table S3.** Computational run-times (sec) of the merging programs. **Table S4.** Memory usage of the merging programs (MB). **Note S2.** Methods for producing a merged read. **Table S5.** Merging schemes of the programs. **Note S3.** notes on the merging programs. **Note S4.** NGmerge-PEAR disagreement. (PDF 786 kb)

Additional file 2: **Table S1.** Details of the 33 datasets downloaded from SRA. The “Number of Sequencing Runs” gives the number of individual SRA “run” datasets that appeared to have been derived from the same sequencing run and thus were concatenated. Multiple datasets from the same SRA study that were not combined were given separate designations (“_0”, “_1”, etc.). The “Instrument” column is based on the metadata given in each SRA dataset and may not be accurate. (XLSX 13 kb)

## Abbreviations

PCR: Polymerase chain reaction
SRA: Sequence Read Archive
