# Supplementary material for: NGmerge: merging paired-end reads via novel empirically-derived models of sequencing errors
Source: BMC Bioinformatics. 2018 Dec 20;19:536. doi: 10.1186/s12859-018-2579-2 (PMC6302405; doi:10.1186/s12859-018-2579-2)
Supplement: Supplementary file 1 — Figure S1. Quality score profiles of the merging programs. Figure S2. PhiX library fragment lengths and paired-end read overlaps. Figure S3. Error rate calculation. Table S2. Error rates before and after merging. Note S1. Benchmarking of merging programs. Table S3. Computational run-times (sec) of the merging programs. Table S4. Memory usage of the merging programs (MB). Note S2. Methods for producing a merged read. Table S5. Merging schemes of the programs. Note S3. notes on the merging programs. Note S4. NGmerge-PEAR disagreement. (PDF 786 kb) [file 12859_2018_2579_MOESM1_ESM.pdf]

NGmerge: merging paired-end reads via novel empirically-derived models of sequencing errors

John M. Gaspar

Supplementary Information

Contents

Figure S1: Quality score profiles of the merging programs 2

Figure S2: PhiX fragment lengths and paired-end read overlaps 3

Figure S3: Error rate calculation 4

Table S2. Error rates before and after merging 5

Note S1: Benchmarking of merging programs 6

Note S2: Methods for producing a merged read 7

Note S3: Additional notes on the merging programs 10

Note S4: NGmerge-PEAR disagreement 11

Figure S1: Quality score profiles of the merging programs

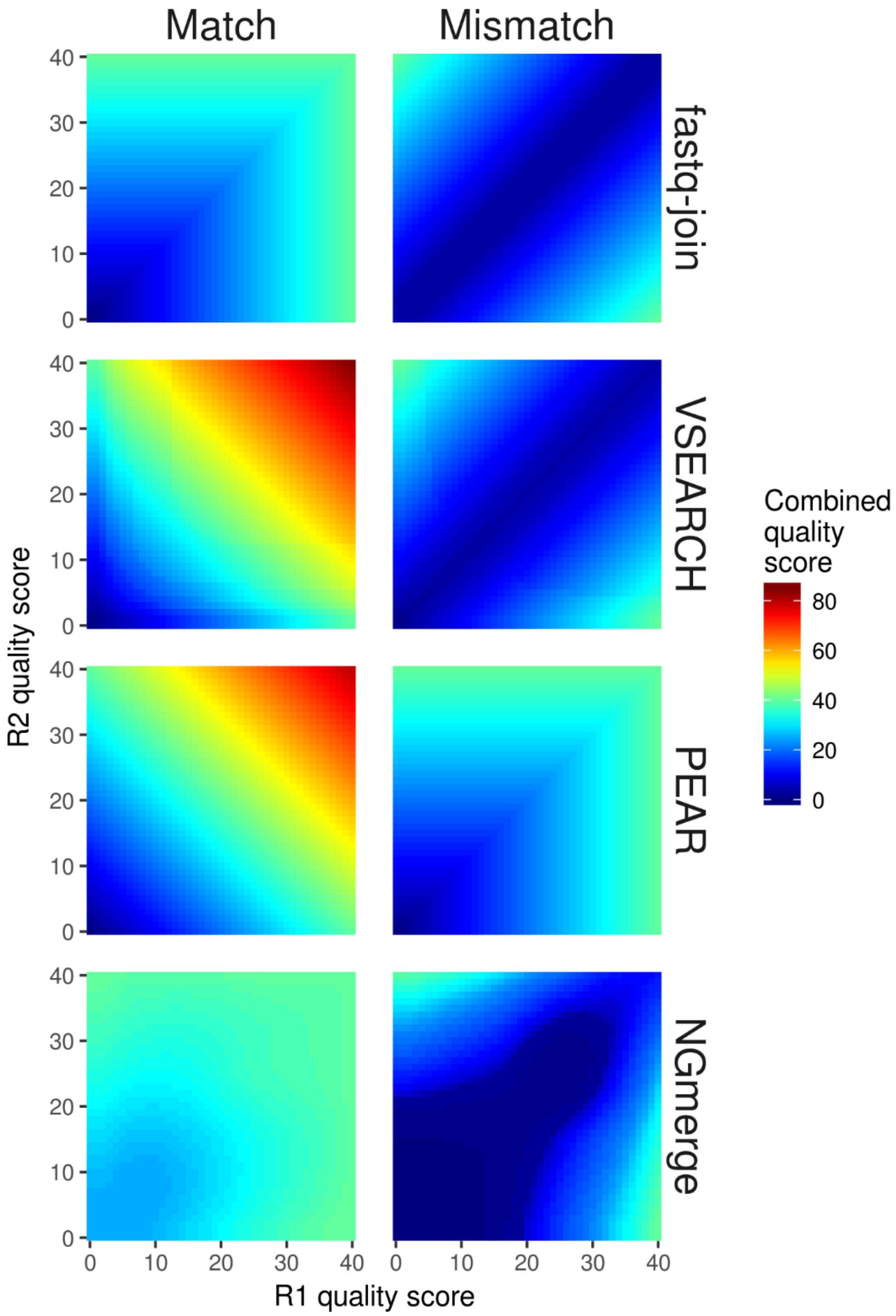

Figure S1. Where a pair of reads (R1, R2) overlap, the merging programs assign quality scores for the merged read based on these profiles, and whether the R1 and R2 bases agree ("Match") or not ("Mismatch"). For additional details, see Table S5 in Note S2.

Figure S2: PhiX fragment lengths and paired-end read overlaps

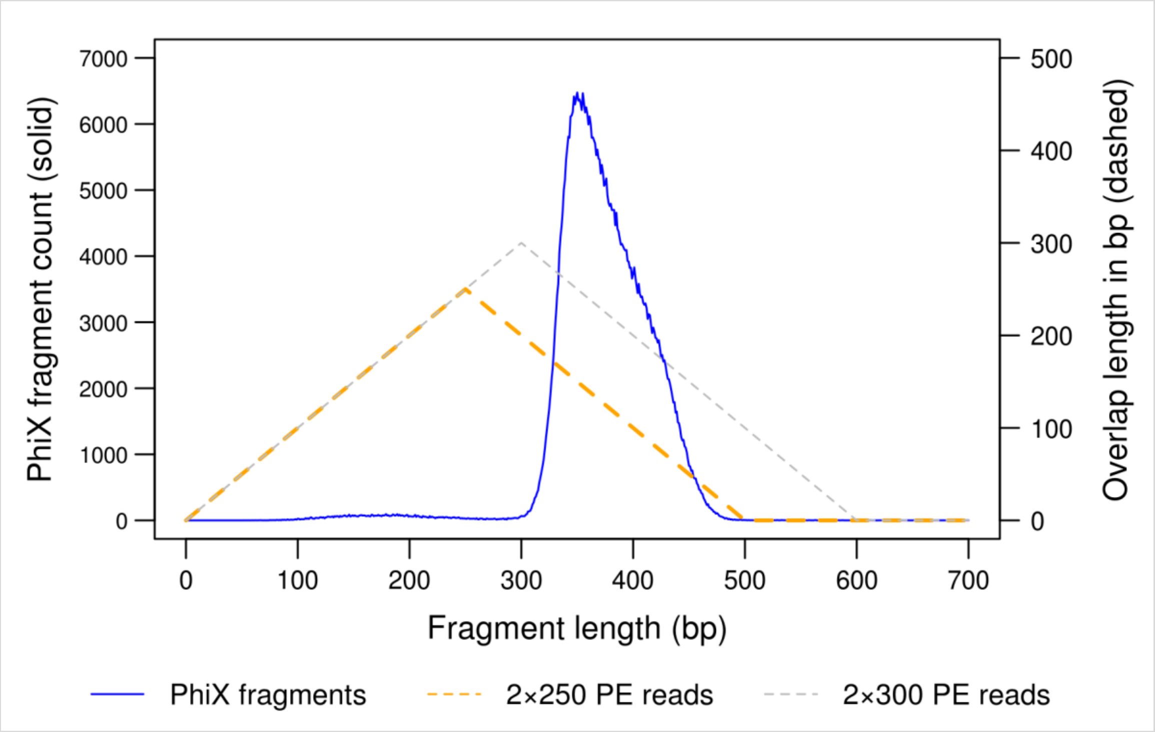

Figure S2. PhiX library fragment lengths and paired-end read overlaps. The PhiX library produced by Illumina has an average fragment length of around 375bp, with most of the fragments between 300bp and 500bp (blue line). With a sequencing run of 2×250bp (orange line), almost all of the paired-end reads derived from PhiX fragments will have some overlap.

Figure S3: Error rate calculation

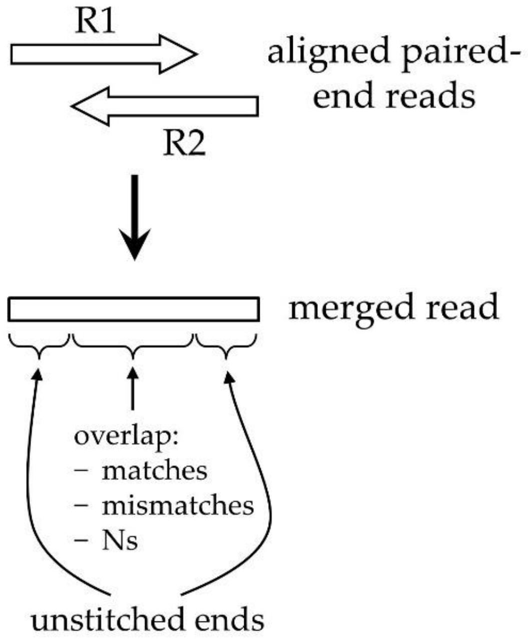

Figure S3. Two paired-end reads are aligned and merged into a single read. The errors of the merged read are tabulated separately for the unstitched ends and for the overlap region. Each base in the overlap is further broken down into one of three categories: where the R1 and R2 bases agree (match), where they disagree (mismatch), and where one (or both) was an ambiguous base (N).

Table S2. Error rates before and after merging

Table S2. Error rates before and after merging. Note that different "Before" values are due to the programs' analyzing slightly different sets of reads (see Table S1).

	Before	After
fastq-join	1.66×10^{-2}	9.56×10^{-4}
VSEARCH	1.95×10^{-2}	1.39×10^{-3}
PEAR	2.54×10^{-2}	1.98×10^{-3}
NGmerge	1.67×10^{-2}	9.25×10^{-4}
NGmerge (-p 0.2)	2.33×10^{-2}	1.69×10^{-3}

Note S1: Benchmarking of merging programs

The reads from three SRA studies were chosen to benchmark the merging programs. Because neither fastq-join nor VSEARCH consider dovetailed alignments, both programs were given reads from which adapters were already removed. All programs were run on a single processor and producing uncompressed output. Reported run-times are the averages of five runs (Table S3).

Table S3. Computational run-times (sec) of the merging programs.

SRA study	Raw reads analyzed	fastq-join*	VSEARCH*	PEAR	NGmerge
SRP059074	11,236	1.3	0.7	16.1	0.7
SRP111842	217,968	21.1	11.1	339.3	16.6
SRP011583	1,322,164	127.4	60.9	2039.3	66.4

* not including the time to trim adapters from the reads

Memory usage was measured using the program valgrind (Table S4). Although both VSEARCH and PEAR use far more memory than fastq-join and NGmerge, this is unlikely to be a significant limitation for most users.

Table S4. Memory usage of the merging programs (MB).

SRA study	Raw reads analyzed	fastq-join	VSEARCH	PEAR	NGmerge
SRP059074	11,236	0.005	35.7	190.7	0.17
SRP111842	217,968	0.005	144.9	190.7	0.17
SRP011583	1,322,164	0.005	146.0	190.7	0.17

Note S2: Methods for producing a merged read

When deciding on a base and quality score for a given position of a merged read from an overlapping pair of reads, the four programs (fastq-join, VSEARCH, PEAR, and NGmerge) utilize different procedures (Table S5; note that FLASH is nearly identical to fastq-join). The non-overlapping ends are left unmodified.

Table S5. Merging schemes of the programs. At a given position, the base and quality score of the merged read depends on comparisons of the bases (R1, R2) and quality scores (Q1, Q2) of the original reads.

base comparison	quality score comparison		Merging program: fastq-join	VSEARCH	PEAR	NGmerge
R1 = R2	any	base	R1	R1	R1	R1
		quality score	max(Q1, Q2)	Q1 + Q2 + ε_{match}(Q1, Q2) *†	Q1 + Q2 *	match matrix ‡
R1 ≠ R2	Q1 > Q2	base	R1	R1	R1	R1
		quality score	max(Q1 - Q2, 3)	Q1 - Q2 + $\varepsilon_{mismatch}$(Q1, Q2) †	Q1	mismatch matrix ‡
	Q1 < Q2	base	R2	R2	R2	R2
		quality score	max(Q2 - Q1, 3)	Q2 - Q1 + $\varepsilon_{mismatch}$(Q1, Q2) †	Q2	mismatch matrix ‡
	Q1 = Q2	base	R2	R2	R2	R1/R2 §
		quality score	3	$\varepsilon_{mismatch}$(Q1, Q2) †	Q1	mismatch matrix ‡

* VSEARCH and PEAR impose maxima on quality scores (by default, 41 and 40, respectively), although these caps can be altered (--fastq_qmaxout and -c arguments, respectively).

† VSEARCH uses schemes based on Edgar and Flyvbjerg (2015). For base matches, the resulting quality score is equivalent to the sum of the original scores plus a small value (ε_{match}) that depends on the original scores and ranges from -1 to +5 (mean: +3.6). For base mismatches, the resulting score is the difference of quality scores plus a small value ($\varepsilon_{mismatch}$), ranging from 0 to +3 (mean: +0.8).

‡ By default, NGmerge uses matrices of empirically-derived values for quality scores. An alternative option (-g) is to follow a scheme similar to that of fastq-join.

§ When the bases disagree with equal quality scores, NGmerge selects the base that is closer to its read's 5′ end.

In terms of base selection for the merged read, the only disagreement occurs when the bases of R1 and R2 disagree but with equal quality scores. In these cases, NGmerge selects the base that is closer to its read's 5′ end, whereas the other three merging programs automatically use the R2 base. NGmerge's approach proved to be correct a

majority of the time with each of the Harvard training datasets, and on average its accuracy was 57.0%. However, it fared worse with the SRA test datasets, being correct only 50.1% of the time. Still, this was better than the other mergers; for example, PEAR’s accuracy in these cases was just 34.2%.

There is further work to be done in understanding the R1/R2 interplay when the quality scores are equal (or close). A consensus-based k-mer approach, such as that implemented in CASPER, is almost certain to perform better in the context of variant-free PhiX reads, but such an approach may have difficulty distinguishing sequencing errors from true biological variants in real samples.

<u>Illustrative example</u>

Consider the following pair of reads:

```
@read 1
CTCACACTCAATCTTTTATCACGAAGTCATGATTGAATCGCGAGTGGTCG
+
1101?B10>F111122BE1B22<EAFC12FB22BFG12>G/<<B>F/11>

@read 2
TTATCGCAATCTGCCGACCACTCGCGCTTTAATCATGACTTCGTGGTAAA
+
;/:00....:000009AA;AE/;E@-----;//;//;EFFB99B91/F/F
```

According to the alignment output of NGmerge, the optimal alignment contained three mismatches:

```
read
seq_R1:  CTCACACTCAATCTTTTATCACGAAGTCATGATTGAATCGCGAGTGGTCG
                       |||| ||||||||||||||| || ||||||||||||
seq_R2:                TTTACCACGAAGTCATGATTAAAGCGCGAGTGGTCGGCAGATTGCGATAA

qual_R1: 1101?B10>F111122BE1B22<EAFC12FB22BFG12>G/<<B>F/11>
qual_R2:               F/F/19B99BFFE;//;//;-----@E;/EA;AA900000:....00:/;
```

All four merging programs agreed on this optimal alignment. Each program produced a slightly different merged read, following the scheme detailed in Table S5:

NGmerge

```
@read
CTCACACTCAATCTTTTATCACGAAGTCATGATTGAATCGCGAGTGGTCGGCAGATTGCGATAA
+
1101?B10>F1111G>HG"GEBFHHHHB>GG>>G?H="DHFFCHGHDDBD0000:....00:/;
```

fastq-join

```
@read 1
CTCACACTCAATCTTTTACCACGAAGTCATGATTGAATCGCGAGTGGTCGGCAGATTGCGATAA
+
1101?B10>F1111F2FE$BB9<EFFE;2FB22B:G1&>GE<<EAFAA9>0000:....00:/;
```

VSEARCH

```
@read 1
CTCACACTCAATCTTTTACCACGAAGTCATGATTGAATCGCGAGTGGTCGGCAGATTGCGATAA
+
1101?B10>F1111JEJJ$JJJJJJJJJEJJEEJ:JA'JJJJJJJJJJJJ0000:....00:/;
```

PEAR

```
@read 1
CTCACACTCAATCTTTTACCACGAAGTCATGATTGAATCGCGAGTGGTCGGCAGATTGCGATAA
+
1101?B10>F1111I@II1IIIIIIIII@II@@IFI=2IIIIIIIIIIII0000:....00:/;
```

The only location where the merged sequences differed occurred at the first mismatch, where the bases (T-C) had equal quality scores ('1'). NGmerge chose the R1 base as the merged base because it was closer to the R1 5′ end than the R2 5′ end, whereas the other programs selected the R2 base automatically. All the programs decreased the quality score to near zero, except for PEAR.

With the other mismatches (G-A and T-G), all four programs selected the base with the higher quality score. To produce the merged quality score, both fastq-join and VSEARCH subtracted the quality scores, and NGmerge reduced the values based on its empirically-derived 'mismatch' matrix. Again, PEAR's merged read had substantially higher quality scores, since it chose the greater of the two reads' quality scores at these positions.

Where the bases of the two reads agreed, VSEARCH and PEAR added the quality scores (with maxima of 41 and 40, respectively), whereas fastq-join selected the maximum of the two scores. NGmerge used its empirically-derived 'match' matrix to assign merged quality scores.

With all four programs, the non-overlapping ends were left unmodified.

Note S3: Additional notes on the merging programs

It is well established that indel errors rarely occur in Illumina sequencing [8]. Hence, the alignment algorithm of NGmerge does not allow gaps (insertions or deletions). The same is true of the other merging programs (fastq-join, FLASH, VSEARCH, and PEAR). One merging program that does allow indels is SeqPrep (github.com/jstjohn/SeqPrep). When analyzing the SRA datasets, SeqPrep aligned just 311 read pairs with gaps. Furthermore, most of the 311 resulting merged reads were mapped to PhiX with an insertion, and the remainder were unmapped or mapped with a series of substitutions near one end that could have been aligned with a gap. Hence, permitting indels in the alignments seems to provide little benefit.

NGmerge can produce output files that are gzip-compressed or not, based on the user's specifications and weighing the value of decreased disk usage versus increased run-time. Fastq-join also produces either type of output. By contrast, the outputs from VSEARCH and PEAR are always uncompressed.

NGmerge is multithreaded using OpenMP 4.0. VSEARCH and PEAR are also multithreaded, whereas fastq-join is not.

Note S4: NGmerge-PEAR disagreement

NGmerge selects an optimal alignment based on the minimal fraction mismatch. By contrast, PEAR uses a statistical framework to judge each potential alignment. Although the two programs usually agree on alignments, the rare cases where they disagree shed light on the differences between their approaches. In particular, PEAR favors alignments with longer overlaps, even with more mismatches.

For example, consider the read pair “SRR5758282.243760” from the SRP110535 dataset in the SRA. NGmerge aligned this pair of reads with a 52bp overlap, no mismatches, and long 3’ overhangs (putative adapters):

```
R1  --------------------------------------------------------------------------------
R2  GAGTGGAAAATAAGTAGGCGGATTGGGATGGTTAAGGGTGAAATGCATAGATATAGAGCAGAACAACGATGTCGAAGGGA

R1  --------------------------------------------------------------------------------
R2  GCGTACTATATTATAGCTGAATCTGAAGCACGAATGCGTGGGTATCAAACTGTTTTTTTTAATGATACGGCGACCACCGA

R1  --------------------------------------TGCGTAACCGTCTTCTCGTTCTCTAAAAACCATTTTTCGTCC
                                          ||||||||||||||||||||||||||||||||||||||||||
R2  GATCTACACTCTTTCCCTACACGACGCTCTTCCGATCTTGCGTAACCGTCTTCTCGTTCTCTAAAAACCATTTTTCGTCC

R1  CCTTCGGGGCAGATCGGAAGAGCGGTTCAGCAGGAATGCCGAGACCGATCTCGTATGCCGTCTTCTGCTTGAAAAAAAAA
    ||||||||||
R2  CCTTCGGGGC----------------------------------------------------------------------

R1  ATGTAAAAGAGGTAAGCGGAATGTGTTTTGTAGCGGTGACATGCATAGATATAACACAGAACCCCGATTCCTAAGGCAGC
R2  --------------------------------------------------------------------------------

R1  TTACTATAATACAACTGACGCTCATGCACGACAGCGTGGGTATCCAAC
R2  ------------------------------------------------
```

PEAR aligned these reads without 3’ overhangs. The 130bp overlapping region had 35 mismatches:

```
R1  TGCGTAACCGTCTTCTCGTTCTCTAAAAACCATTTTTCGTCCCCTTCGGGGCAGATCGGAAGAGCGGTTCAGCAGGAATG
R2  --------------------------------------------------------------------------------

R1  CCGAGACCGATCTCGTATGCCGTCTTCTGCTTGAAAAAAAAAATGTAAAAGAGGTAAGCGGAATGTGTTTTGTAGCGGTG
                                             | || |||| | ||| ||||| || | |   ||  ||||
R2  ----------------------------------------GAGTGGAAAATAAGTAGGCGGATTGGGATGGTTAAGGGTG

R1  ACATGCATAGATATAACACAGAACCCCGATTCCTAAGGCAGCTTACTATAATACAACTGACGCTCATGCACGACAGCGTG
    | |||||||||||||   ||||||  ||||  | |||| ||| ||||||| || | ||||  || | ||||||  |||||
R2  AAATGCATAGATATAGAGCAGAACAACGATGTCGAAGGGAGCGTACTATATTATAGCTGAATCTGAAGCACGAATGCGTG

R1  GGTATCCAAC----------------------------------------------------------------------
    |||||| |||
R2  GGTATCAAACTGTTTTTTTTAATGATACGGCGACCACCGAGATCTACACTCTTTCCCTACACGACGCTCTTCCGATCTTG

R1  --------------------------------------------------
R2  CGTAACCGTCTTCTCGTTCTCTAAAAACCATTTTTCGTCCCCTTCGGGGC
```

With NGmerge, the resulting 52bp merged read was mapped by Bowtie2 to PhiX (position 4330) with no errors. Furthermore, both 3′ overhangs matched the canonical Illumina adapter sequence (AGATCGG...). By contrast, the 370bp merged read produced by PEAR was unmapped to PhiX. Thus, NGmerge appeared to produce the correct result.

Of all the reads in the 33 SRA datasets analyzed, there were just 282 read pairs that NGmerge and PEAR aligned differently, like this example. However, in the sequencing of DNA fragments containing more repetitive sequences than what is found in the PhiX genome, we would expect this type of discrepancy to occur more frequently.

Another difference between PEAR and the other merging programs is that the former is more aggressive in forcing reads together. So, although it produced the greatest number of merged reads aligning to PhiX (in total, 2.1% more than NGmerge [Table S1]), it also had 2.6 times as many reads that were successfully merged but remained unmapped to PhiX (including the example read above). Those who desire an aggressive merging algorithm but are wary of the false positives produced by PEAR may consider increasing the fraction mismatch parameter of NGmerge. For example, allowing 20% mismatches (-p 0.2) produced a 3.4% increase in the number of reads aligning to PhiX (Table S1), but also a higher post-merging error rate, similar to that seen with PEAR (Fig. 4A).
